# Impacts of COVID-19 Pandemic on Dietary Consumption among Chinese Residents: Evidence from Provincial-Level Panel Data

**DOI:** 10.3390/ijerph19137612

**Published:** 2022-06-22

**Authors:** Xiaodong Zheng, Yinglin Wang, Yue Zhang, Tinghe Deng, Yuanzheng Yang

**Affiliations:** 1 School of Economics, Zhejiang Gongshang University, Hangzhou 310018, China; yinglinwang99@163.com (Y.W.); zhangyuezjsu@163.com (Y.Z.); 2 Chinese Center for Health Education, Beijing 100011, China; dengtinghe1988@163.com; 3 Rural Development Institute, Chinese Academy of Social Science, Beijing 100732, China; yangyz@cass.org.cn

**Keywords:** COVID-19, dietary consumption, nutritional health, health awareness, China

## Abstract

The COVID-19 pandemic has profoundly affected people’s daily lives, including their dietary behaviors. Using a panel data set of 31 provinces from 2015 to 2020, this study employed two-way fixed effects (FE) models to examine the impacts of the COVID-19 pandemic on dietary consumption among Chinese residents. The results showed that the COVID-19 pandemic positively affected residents’ consumption of grain, eggs, dairy, and white meat (poultry and aquatic products), while it had a negative effect on individuals’ red meat consumption in both urban and rural areas. These results were robust to different measures of the COVID-19 pandemic, including the number of confirmed cases, suspect cases, and dead cases. Comparatively, the changes in food consumption induced by the COVID-19 pandemic were more prominent for Chinese residents who lived in rural areas than urban areas. In addition, compared to their counterparts, the dietary consequences of the pandemic were more pronounced for residents living in the eastern region and regions with a high old-age dependency ratio and low illiteracy rate. Furthermore, the estimation results of the quantile regression model for panel data suggested that the COVID-19 pandemic had relatively larger impacts on the dietary consumption of Chinese residents at lower quantiles of food consumption compared with those at higher quantiles. Overall, the results of this study suggested that Chinese residents had a healthier diet after the outbreak of the COVID-19 pandemic. We discussed possible mechanisms, including health awareness, income, food supply and prices, and other behavioral changes during COVID-19 (e.g., physical activity and cooking). To further improve residents’ dietary behaviors and health, our study proposed relevant measures, such as increasing residents’ dietary knowledge, ensuring employment and income, and strengthening the food supply chain resilience during the pandemic.

## 1. Introduction

The sudden and widespread coronavirus disease (COVID-19) pandemic has had a wide-ranging impact on the social economy and people’s daily lives worldwide. According to the statistics reported by World Health Organization (WHO), as of 19 April 2022, there have been more than 0.5 billion confirmed cases of COVID-19 globally, including more than a million deaths. In China, from 3 January 2020 to 19 April 2022, there were 979,966 confirmed cases of COVID-19, with 14,661 deaths. The faster than expected spread of the COVID-19 pandemic has seriously affected the prospects of global economic growth and the confidence of all parties [1]. Meanwhile, the COVID-19 pandemic has also widely affected individuals’ lifestyle and health behaviors, such as physical activity and sedentary time [2], social activity and leisure time [3,4], and sleep problems [5,6]. Although a few studies have investigated the effects of the COVID-19 pandemic on people’s dietary behaviors in developed countries [7,8], research regarding the effects of the pandemic on the dietary consumption of residents in developing countries like China is still limited. Given that dietary consumption is closed correlated to diseases (e.g., cardiovascular disease and cancer) and mortality risk [9], it is of considerable importance to examine the dietary consequences of the COVID-19 pandemic in China. The findings also have crucial implications for developing countries or regions with similar social contexts.

There are a variety of determinants of people’s dietary consumption, such as food supply, food price, socioeconomic factors (e.g., family size and income), individuals’ preferences, and health knowledge [10,11,12]. COVID-19 has been a new driving factor of individuals’ dietary behaviors after the pandemic outbreak. Theoretically, the COVID-19 pandemic can affect dietary consumption among Chinese residents in the following ways. First, the impact of the COVID-19 pandemic on food supply cannot be ignored. In order to limit the spread of the COVID-19 pandemic and ensure the safety of people’s lives and property, the Chinese government has taken relatively strict control measures to restrict population movement and create roadblocks [13,14]. If an outbreak of the COVID-19 pandemic occurs somewhere, the government will close public places and restrict people’s movement to prevent the spread of the disease caused by crowds gathering together [15]. These restrictions have prevented the COVID-19 virus from spreading widely, but they have also seriously affected the industries of grain planting, animal husbandry, agricultural product processing, and food logistics [16]. The international COVID-19 pandemic is also worrying, with several categories of food imports decreasing to a certain extent [17]. As a result, these measures negatively influence the amount of domestic food supply, which could reduce the quantity of residents’ food consumption and the diversity of food choices.

Second, as domestic demand declines due to the COVID-19 pandemic struck, a large number of residents are at risk of losing their jobs. Meanwhile, the delayed resumption of work reduces the effective working hours of most workers, which leads to a reduction in family income [18]. The decline in income can further strengthen the budgetary constraints faced by residents, reduce their capability to purchase high-quality food, and subsequently have a negative effect on the dietary patterns of residents, including quantity, quality, and diversity of food consumption [19].

Third, the COVID-19 pandemic may increase uncertainty about the future and change residents’ dietary behaviors due to their risk preferences. When residents face sudden public health events and under the situation of incomplete information, for rational consideration, they may increase their current healthy consumption to improve their ability to resist risks [20,21]. The most convenient way of healthy consumption is to adjust the dietary pattern and increase food consumption, especially healthy food with high protein and rich micronutrients.

In addition, residents may also tend to have a more balanced diet due to some behavioral changes during the pandemic. For example, given that the COVID-19 pandemic results in constraints on physical activities due to the lockdown [22], people may adjust their dietary behavior as an alternative measure to maintain physical fitness. Evidence has also shown that households have more careful food planning and management since they have to stay and cook at home during the lockdown [23]. As such, people might also pay more attention to the nutritional values of the food they cook at home.

Overall, according to the aforementioned mechanisms underlying the relationships between the COVID-19 and people’s dietary consumption, it still remains an open question of the direction and magnitude of the pandemic’s impacts on dietary consumption. However, it is clear that the COVID-19 pandemic could have positive dietary effects on residents who have suffered relatively mild impacts of the pandemic on wage income, food supply, population mobility, and those who have significantly increased their consciousness of a healthy diet.

Up to the present, a few studies have examined the impact of the COVID-19 pandemic on food consumption in China. However, past research has mainly focused on a specific type of food consumption or a specific region (province) [24,25]. Different from previous studies, this study aims to examine the dietary consequences of the COVID-19 pandemic among Chinese residents, focusing on a variety of foods and using a nationally representative sample. Specifically, based on the provincial-level panel data from 2015 to 2020, this study employed two-way fixed effects (FE) models to examine the impacts of the COVID-19 pandemic on eight categories of food consumption. Further, we also investigated the heterogeneous effects of the pandemic on residents’ dietary consumption in urban–rural areas, different regions, different proportions of older adults, and different education levels. The results showed that the COVID-19 pandemic has increased Chinese residents’ consumption of healthy food, such as eggs and dairy products. The pandemic has negatively affected people’s consumption of red meat (with high-fat content). In addition, the positively dietary effects of the COVID-19 pandemic were more prominent for residents living in rural areas, eastern regions, regions with a high old-age dependency ratio and a low illiterate rate, and those who consumed a low level of food.

This study contributes to the literature by investigating the impacts and heterogeneity of the COVID-19 pandemic on residents’ dietary consumption in China. The findings can not only shed light on the changes in lifestyle behaviors during the pandemic, but also provide policy implications to improve public health by implementing evidence-based interventions to promote a healthy diet, especially for residents who are lack health knowledge and financially vulnerable.

The rest of this article is organized as follows. Section 2 describes the data and methods and reports the descriptive statistics. Section 3 presents the main results of the empirical analysis. Section 4 discusses the results, and Section 5 gives the conclusions of this study.

## 2. Methods

### 2.1. Data

The data regarding residents’ dietary consumption and socioeconomic characteristics were from the *China Statistical Yearbook* (CSY) released by the National Bureau of Statistics of China (NBSC). NBSC undertakes household surveys with more than 160,000 households scattered in China every year, which makes the survey nationally representative [26]. The survey contents include household demographic characteristics, income, consumption, and other information. We used the aggregated data on dietary consumption from the CSY. The major foods investigated in this study include grain, vegetables, red meat, poultry, aquatic products, dairy, and fruits. Each category of food consumption was aggregated at the provincial level for urban and rural households separately by NBSC. Given the unavailability of food consumption statistics by region in CSY 2015 and before, we used CSY from 2016 to 2021, which recorded provincial-level data from 2015 to 2020. The data regarding the COVID-19 pandemic included the number of confirmed cases, suspect cases, and dead cases. They were hand-collected from the official websites of the health commission of each province in China. Since the latest year of provincial-level data used in this study was 2020, the COVID-19 pandemic data were updated to 30 December 2020. After merging these two datasets, the final sample for our empirical analysis included a total of 186 observations, consisting of 31 provinces in mainland China from 2015 to 2020.

### 2.2. Measures

The dependent variable of this study was the dietary consumption of Chinese residents. Given the data availability, this study mainly focused on the consumption quantity of different categories of foods rather than particular types of nutrition intake. Specifically, the major foods studied in our study include grains (e.g., rice, wheat-based food, coarse cereals, beans), red meat (e.g., beef, lamb, pork), poultry (e.g., chicken, duck, goose), aquatic products (e.g., fish, shellfish, shrimp), eggs (e.g., duck, chicken or quail eggs), dairy (e.g., milk, yogurt, cheese), vegetables (e.g., light vegetables, dark vegetables), and fruits (e.g., apples, bananas, strawberries). These food categories are also the key elements of food groups to measure dietary diversity according to the guidelines of the Food and Agriculture Organization of the United Nations (FAO) [27,28]. The consumption of the above eight food categories was measured by per capita consumption (in kilograms) within a given province.

The key independent variable of this study was the COVID-19 pandemic. We mainly used the number of the confirmed COVID-19 cases within a province to measure the severity of the pandemic. A confirmed case was defined as an individual who had a confirmatory viral test, and that specimen tested positive for SARS-CoV-2, which is the virus that caused the COVID-19 pandemic. In addition, as a robustness check, we also used other proxy variables of the COVID-19, including the number of suspect cases and dead cases. Among them, a suspect case of COVID-19 refers to someone who has symptoms of coronavirus, who has been tested or is about to be tested but has not found out the results of the tests yet. A dead case denotes that COVID-19 is the “probable” or “presumed” cause of someone’s death. The confirmed, suspect, and dead counts of COVID-19 were aggregated at the provincial level.

To reduce estimation bias due to confounding heterogeneity, we controlled for a number of regional characteristics as covariates in our regression analyses. Given the availability of data, the empirical analysis mainly controlled for factors related to food production and supply, household socioeconomic status, and family structure. These factors were shown to be crucial determinants of people’s dietary behaviors [10,11,12]. Specifically, the covariates included proportion of the primary industry (%), per capita GDP (in logarithm form), child dependency ratio (number of children aged 0 to 14 years per 100 persons aged 15 to 64 years, %), old-age dependency ratio (number of older adults aged 65 years or over per 100 persons aged 15 to 64 years, %), and illiteracy rate (percentage of the illiterate population to total population aged 15 and over, %). We also controlled for provincial fixed effects and year fixed effects to reduce omitted variable bias due to potential unobserved factors.

### 2.3. Statistical Analysis

The statistical analysis of this study was composed of four parts, including descriptive statistics, baseline regression analysis, heterogeneity analysis, and robustness checks. First, descriptive statistics were computed for all variables used in this study. In the meantime, we also employed two-tailed *t*-tests to compare the differences in food consumption and covariates before and after the outbreak (year 2020) of the COVID-19 pandemic. Second, we employed two-way fixed effects models to examine the impacts of the COVID-19 pandemic on dietary consumption among Chinese residents. Although the pandemic was assumed to be exogenous, controlling for provincial fixed effects and year fixed effects could better rule out the estimation bias due to potential unobserved heterogeneity. In particular, the fixed effects models were specified as follows:

(1) $$Y_{it}=\beta_{0}+\beta_{1}Covid_{it}+\Gamma X_{it}+\sigma_{i}+\lambda_{t}+\varepsilon_{it}$$

where $Y_{it}$ represents the outcome variable of a specific category of food consumption for the *i*th province in year *t* (*t* ranges from 2015 to 2020). $Covid_{it}$ denotes the proxy variable of the COVID-19 pandemic (e.g., number of confirmed cases). $X_{it}$ represents a series of covariates. $\sigma_{i}\;$and $\lambda_{t}$ represent provincial and year fixed effects, respectively. $\varepsilon_{it}$ is the error term. To allow for regional correlations, standard errors were clustered at the provincial level. Given that the measures of food consumption and the COVID-19 pandemic were continuous variables, we took the natural logarithm form of these variables to facilitate the interpretation of the estimated results. Similarly, continuous variables in covariates were also taken as natural logarithm forms. The estimated coefficients of $Covid_{it}$ ($\beta_{1}$) and its significance level are the main focus of this study.

Third, to shed light on the heterogeneity of the pandemic’s impacts on dietary consumption, we conducted a stratified analysis through estimating fixed effects models for subgroups by urban–rural area, region, old-age dependency ratio, and illiteracy rate. To a certain extent, the selection of these segments of population is conducive to indirectly revealing the mechanisms behind the relationship between the COVID-19 pandemic and dietary consumption and developing targeted solutions to improve residents’ health. On the one hand, the economic development level, food supply capacity, and residents’ dietary habits are different between rural and urban areas and among different regions. They are also the potential reasons to explain the dietary consequences of the COVID-19 pandemic. On the other hand, given that older adults are at a higher health risk than younger ones during the pandemic [29,30], the heterogeneous impacts of COVID-19 by the old-age dependency ratio can be partly attributed to the changes in health consciousness, which is also a crucial mechanism underlying the dietary effects of the pandemic. In addition, note that human capital (e.g., education and cognitive skills) plays a crucial role in behavioral outcomes [31], investigating the heterogeneous effects by different education levels is helpful to propose corresponding implications for policy improvement.

Specifically, we divided 31 provinces into three regions according to the standard of NSBC, where the eastern region includes 11 provinces or municipalities (Beijing, Tianjin, Hebei, Liaoning, Shanghai, Jiangsu, Zhejiang, Fujian, Shandong, Guangdong, and Hainan), the central region includes 8 provinces (Shanxi, Jilin, Heilongjiang, Anhui, Jiangxi, Henan, Hubei, and Hunan), and the western region consists of 12 provinces or autonomous regions or municipalities (Mongolia, Guangxi, Chongqing, Sichuan, Guizhou, Yunnan, Shaanxi, Gansu, Qinghai, Ningxia, Tibet, and Xinjiang). In terms of different levels of old-age dependency ratio and illiteracy rate, we used the median as a grouping criterion to distinguish between high-level and low-level groups. In addition, we employed the quantile regression model for panel data to explore the heterogeneous effects of the COVID-19 pandemic on different percentiles of food consumption rather than average effects. Such a method allows for the estimation of heterogeneous effects throughout the conditional distribution of our dependent variable (food consumption) while controlling for provincial and time-specific confounders [32]. The quantile regression for panel data used in this study was specified as follows:

(2) $$Q^{\theta }(Y_{it})=\beta_{0}^{\theta }+\beta_{1}^{\theta }Covid_{it}^{\theta }+\Gamma^{\theta }X_{it}+\sigma_{i}+\lambda_{t}+\varepsilon_{it}$$

where θ (0<θ<1) is a given quantile and $Q^{\theta }(Y_{it})$ represents the conditional distribution of a specific food consumption category on the θth quantile. $\beta_{1}^{\theta }$ denotes the impact of the pandemic on food consumption on the θth quantile. In practice, we set five quantiles to investigate the heterogeneous effects of the pandemic on different distributions of dietary consumption, including 10th, 30th, 50th, 70th, and 90th quantiles. If the marginal effects of the COVID-19 were larger for low percentiles (e.g., 10th quantile) than that for high percentiles (90th quantile), it suggests that the effect size of the pandemic on food consumption was larger for those who consumed lower levels of food.

Fourth, to check the robustness of our main findings, we used proxy variables of the COVID-19 other than the number of confirmed cases within each province. In particular, we used the suspect counts and dead counts of COVID-19 as alternative measures of the pandemic, respectively. We then compared the regression estimates using different measures of the COVID-19 pandemic to examine whether our results were robust. In addition, as another robustness check, we further controlled for specific food price indexes to account for impacts of price variations due to the pandemic.

## 3. Results

Table 1 presents the descriptive characteristics of the full sample and two subgroups defined by the timing of the outbreak of the COVID-19 pandemic (i.e., 2015~2019 and 2020). The results showed that most categories of food consumption among urban and rural residents increased after the outbreak of the COVID-19 pandemic. Without controlling for covariates, the per capita consumption of grain, poultry, and eggs was significantly larger in 2020 than the average consumption from 2015 to 2019, respectively. Specifically, the consumption of grain, poultry, and eggs increased by 6.2% (*p* < 0.05), 19.9% (*p* < 0.05), and 18% (*p* < 0.01) for urban residents and 7.2% (*p* < 0.05), 34.6% (*p* < 0.01), and 25.1% (*p* < 0.01) for rural residents, respectively. Comparatively, the changes in these food consumptions were more pronounced for rural areas than urban areas. The results of the group comparison also showed that the red meat consumption for both urban and rural areas in 2020 was lower than the average consumption from 2015 to 2019, though it was not statistically significant. The results also showed significant between-group differences in per capita GDP (*p* < 0.1), child dependency ratio (*p* < 0.1), and old-age dependency ratio (*p* < 0.01).

Given that the differences in dietary consumption before and after the COVID-19 pandemic might be confounded by time and regional factors, we implemented a multiple regression analysis controlling for provincial fixed effects and year fixed effects and related covariates (provincial characteristics). The regression estimates of the two-way fixed effects models are shown in Table 2. In Panel A, it can be found that the COVID-19 pandemic had significant positive impacts on the consumption of grain (*p* < 0.01), poultry (*p* < 0.05), eggs (*p* < 0.01), and dairy (*p* < 0.05) in urban areas. In contrast, it had a significant negative effect on red meat consumption (*p* < 0.05). Specifically, for every 100% increase in the confirmed counts of COVID-19, the urban consumption of grain, poultry, eggs, and dairy increased by 1.5%, 1.6%, 2.2%, and 1.0% respectively, whereas the red meat consumption decreased by 1.1%. In terms of food consumption in rural areas, the results showed that the pandemic significantly increased rural consumption of grain (*p* < 0.01), poultry (*p* < 0.01), aquatic products (*p* < 0.01), eggs (*p* < 0.01), and dairy (*p* < 0.05) by 2.2%, 2.8%, 1.3%, 2.9%, and 2.0%, respectively, when the number of confirmed cases increases by 100%. Meanwhile, the pandemic also negatively affected the consumption of red meat (−1.7%, *p* < 0.1) among rural residents. Comparatively, the effect size of COVID-19 on residents’ food consumption was more prominent in rural areas than in urban areas.

Table 3 reports the estimates of fixed effects models for urban and rural residents in the eastern, central, and western regions, respectively. In terms of urban areas, the results in Panel A demonstrated that the COVID-19 pandemic had significant and positive impacts on the residents’ consumption of grain (*p* < 0.01), poultry (*p* < 0.01), eggs (*p* < 0.01), and dairy (*p* < 0.01) in the eastern region of China, while it was not significantly associated with most categories of food consumption in the central and western regions. Similarly, the estimates in Panel B showed that the pandemic positively affected rural residents’ consumption of grain (*p* < 0.01), poultry (*p* < 0.01), eggs (*p* < 0.01), dairy (*p* < 0.05), and vegetables (*p* < 0.01) in the eastern region, while it negatively influenced red meat consumption (*p* < 0.01). As for the central and western regions, the proxy variable of the COVID-19 pandemic was not significant for most categories of food consumption. Comparatively, the marginal effects of the pandemic on residents’ dietary consumption were larger for rural areas than urban areas, which was consistent with our baseline regressions. Overall, the results of stratified analysis by region suggested that the impacts of the COVID-19 pandemic on people’s dietary consumption (no matter positive or negative) were more pronounced for residents who lived in the eastern region than those in the central and western regions.

Table 4 presents the estimated results for subgroups defined by high-level (above the median) and low-level (below the median) old-age dependency ratios for urban areas and rural areas, respectively. In Panel A, the estimated results suggested that the pandemic was significantly associated with most categories of food consumption in urban areas with a high old-age dependency ratio, including increasing consumption of grain (*p* < 0.01), poultry (*p* < 0.01), eggs (*p* < 0.01), dairy (*p* < 0.01), vegetables (*p* < 0.01), and fruits (*p* < 0.1) and reducing red meat consumption (*p* < 0.01). For urban areas with a low old-age dependency ratio, however, the pandemic was not statistically significant for all categories of food consumption in this study. In Panel B, the results also showed that the COVID-19 pandemic had more prominent impacts on rural residents’ food consumption in regions with a high old-age dependency ratio than regions with a low proportion of old adults. Specifically, the proxy variable for the COVID-19 pandemic was significant for grain (*p* < 0.01), red meat (*p* < 0.01), poultry (*p* < 0.01), eggs (*p* < 0.01), and vegetables (*p* < 0.05) for rural residents living in regions with high old-age dependency ratio, while it was only significant for grain (*p* < 0.01) and poultry (*p* < 0.01) with smaller effect sizes for rural areas with low old-age dependency ratio.

Table 5 shows the estimated results of the heterogeneity analysis by illiteracy rate (above or below the median), which were helpful in exploring the differences in responses to dietary consumption between high-level and low-level education. The results in Panel A demonstrated that the COVID-19 pandemic was not statistically significant for all the eight categories of food consumption in urban areas with high illiteracy rates, whereas it was significantly associated with urban residents’ consumption of grain (*p* < 0.01), red meat (*p* < 0.01), poultry (*p* < 0.01), eggs (*p* < 0.01), and dairy (*p* < 0.01) in urban regions with low-illiteracy rate. Similar results were found in rural areas. As shown in Panel B, the pandemic had positive effects on rural residents’ consumption of grain (*p* < 0.01), poultry (*p* < 0.01), aquatic products (*p* < 0.05), and eggs (*p* < 0.01), and negative impacts on red meat (*p* < 0.01) in regions with a low illiteracy rate, while it was only positively linked to grain (*p* < 0.1) and poultry (*p* < 0.01) with relatively small effect sizes in regions with a high illiteracy rate. Therefore, it can be found that the impacts of the pandemic on people’s dietary consumption, including the quantity and diversity of food consumption, were more salient for regions with low illiteracy rates in comparison with those with high illiteracy rates.

Table 6 presents the estimated results of quantile regressions for panel data, controlling for provincial fixed effects and year fixed effects, as well as the aforementioned covariates. Generally, the results in Panel A for urban areas and Panel B for rural areas were consistent. Although the differences between different quantiles of food consumption were not very prominent, the results demonstrated that the COVID-19 pandemic had slightly larger impacts on residents’ food consumption with low quantiles (e.g., 10th quantile) than that with high quantiles (e.g., 90th quantile). Specifically, for urban residents, the pandemic had larger positive effects on people’s consumption of grain, poultry, eggs, and dairy for those who previously consumed relatively low levels of the corresponding foods. Similarly, the COVID-19 pandemic was also more effective in increasing the consumption of grain, poultry, aquatic products, eggs, and dairy for rural residents who had low levels of consumption of these food categories. As such, the results of quantile regressions for panel data suggested that the pandemic’s effects on people’s diets were larger for those who had low levels of food consumption than high levels. This implies that there was a diminishing marginal effect of the impacts of the pandemic on people’s dietary consumption.

The proxy variable for COVID-19 was the confirmed counts in the above empirical analysis. Table 7 shows the results of robustness checks using alternative measures of the COVID-19 pandemic, including the number of suspect cases and dead cases. The estimated results were consistent with our main findings. Specifically, the results in Panel A demonstrated that the pandemic positively affects a number of categories of urban residents’ food consumption, especially grain, poultry, and eggs. The results also demonstrated that the pandemic positively influenced residents’ consumption of dairy and vegetables, depending on the COVID-19 proxy considered. In panel B, it can be found that the alternative proxy variables of the COVID-19 pandemic were positively significant for grain, poultry, aquatic products, eggs, and dairy, and negatively significant for red meat. Comparatively, the effect sizes of the pandemic on people’s dietary consumption were larger for rural residents than urban ones.

To account for the variations in food prices due to the COVID-19 pandemic, we further controlled for the food price variable as another robustness check. The food prices were measured using the price index of each category of food examined in our study, which was drawn from the CSY released by NBSC. Appendix A Table A1 presents the estimation results. Once again, the results on the dietary consequences of the COVID-19 pandemic were consistent with our baseline regressions. Comparatively, the positive effects of the pandemic on residents’ dietary consumption were slightly larger when taking food price into account, suggesting that food price changes could be a potential channel underlying the relationship between the COVID-19 pandemic and residents’ dietary consumption.

## 4. Discussion

Using the provincial-level panel data from 2015 to 2020, which is a national representative sample released by NBSC, this study employed two-way fixed effects models to investigate the impacts of the COVID-19 pandemic on dietary consumption among Chinese residents. The confirmed counts of COVID-19 were used as the proxy variable for COVID-19. We also conducted heterogeneity analysis by comparing the significant levels and effect sizes of the proxy variable of the COVID-19 pandemic in different subgroups defined by region, old-age dependency ratio, and illiteracy rate. Further, the quantile regression for panel data was applied to examine the heterogeneous effects throughout the conditional distribution of variables regarding food consumption. Finally, instead of using confirmed counts of COVID-19 within a province, we checked the robustness of our main findings using suspect counts and dead counts as alternative measures of the pandemic. As another robustness check, we further controlled for food prices to test the validity of our main findings.

This study found that the COVID-19 pandemic had significant and positive impacts on urban and rural residents’ consumption of grain, poultry, eggs, and dairy, whereas it adversely affected red meat consumption. The results were consistent with a previous study using a survey sample in Jiangsu province, which also found the pandemic increased some categories of food consumption [25]. One possible reason is that residents may be inclined to consume more food to increase their viability, which is considered a countermeasure to deal with the risk of uncertainty after the outbreak of the COVID-19 pandemic. Meanwhile, people might also pay more attention to a healthy diet, which is beneficial to improving health status and strengthening the body’s immune system. For example, grain products (e.g., wholegrain foods) are a good source of energy, essential fiber, calcium, iron, and vitamins [33]. Dairy products and eggs are proven to be protein-rich foods, which also deliver concentrated amounts of nutrient vitamins and minerals [34,35]. Poultry meat and aquatic foods also provide high-quality protein and micronutrients with low-fat content [36,37]. The promotion of health awareness could also be a feasible explanation of the negative association between the pandemic and red meat consumption in this study. Because red meat is a category of food with high-fat content, it can lead to cardiovascular disease with excessive consumption, especially for middle-aged and older adults [38]. It is also possible that people have to stay at home from work and earn less income due to the pandemic [39]. Moreover, there could also be a supply shortage for some categories of food due to disruptions to transportation networks and labor shortages [40], which subsequently increases food prices. As such, residents may not be able to afford the consumption of high-price food (e.g., red meat) and increase the consumption of other food instead, such as grain, eggs, and dairy products. In addition, another probable reason is that the COVID-19-induced lockdown adversely affected residents’ physical activity and increased the frequency of cooking meals at home [23,41]. People may, in turn, be more concerned about the nutritional values of the food they cook and develop healthier eating habits to improve their health.

In terms of the heterogeneous impacts of the COVID-19 pandemic on dietary consumption, we found that the dietary consequences of the pandemic (no matter positive or negative) were more pronounced for rural residents compared to their urban counterparts. We interpreted such a finding as a result of the urban–rural difference in the changes in health consciousness regarding food consumption after the outbreak of the COVID-19 pandemic. Past research has shown that urban residents consumed more healthy food (e.g., milk and dairy products) than their rural peers, possibly due to the urban–rural disparities in health awareness, income, and accessibility to such foods [32]. Thus, compared to their urban counterparts, rural residents could increase health consciousness more significantly and gain more health knowledge through public media after the outbreak of the pandemic. In addition, given that rural residents consumed less healthy food than urban ones, the marginal effect sizes of the pandemic on people’s dietary consumption might be larger for rural residents in comparison with their urban peers. This explanation is also supported by our results of quantile regressions for panel data, which suggested that the COVID-19 pandemic had more positive effects on residents who had low levels of food consumption. That is, the effect sizes of COVID-19 on people’s dietary consumption were diminishing with the increase in food consumption, and people with low levels of healthy food consumption were more prone to be affected by the pandemic.

Our heterogeneity analysis also indicated that the impacts of the pandemic on people’s dietary consumption were more prominent in the eastern region than central and western regions. One plausible reason is that, compared to those living in underdeveloped areas, residents in developed regions were less likely to confront food shortages and serious inflation due to a disrupted food supply chain. In other words, developed regions had more complete logistics and retail systems to ensure sufficient food supply and stabilize food prices under emergency circumstances [42]. Hence, the positive effects on health consciousness were more salient for residents living in the eastern region than those in the central and western areas. In addition, we also found heterogeneous effects of the pandemic on people’s dietary consumption by old-age dependency ratio and illiteracy rate. To be specific, the impacts of the pandemic on people’s food consumption were larger for the regions with a high old-age dependency ratio and a low illiteracy rate. A reasonable explanation is that the increase in consciousness of a healthy diet was more prominent for older adults and people with high levels of education after the outbreak of the COVID-19 pandemic. Elderly people are more vulnerable to have serious illnesses during the pandemic, whereas highly educated people are more sensitive and better at adjusting their dietary behaviors to the changes in the public health environment [43,44,45,46].

Our findings have several implications for policy improvement. First, although this study found positive impacts of the pandemic on dietary consumption among Chinese residents, the effect sizes were relatively small. Meanwhile, some categories of food consumption (e.g., vegetables and fruits) were not significantly affected in the full sample. Therefore, the promotion and universal education on a healthy diet are still needed, especially for residents with low levels of education. Many publicity measures are encouraged, such as posting announcements for a healthy diet in public places (e.g., billboards in neighborhoods and bus stations) and organizing public lectures on knowledge of a healthy diet. Second, relevant actions should be implemented to reduce the unemployment rate during the pandemic and ensure residents’ jobs and income for a basic livelihood, particularly in underdeveloped regions with a large number of residents living in relative poverty. In addition, for the vulnerable population such as older adults, targeted cash transfer programs are needed to be implemented if government financial resources permit. Third, it is also necessary to strengthen the construction of logistics systems and warehousing infrastructure (especially in less developed regions), which is crucial to juggling the logistics of supplying food.

This study also has some limitations due to data constraints. First, although our study focused on a variety of food categories, we may not have fully uncovered the relationships between the COVID-19 pandemic and all types of food consumption. In addition, we only investigated the quantity of different categories of food consumption, the diversity and quality of dietary consumption were not discussed in depth due to the lack of relevant data. Second, although we indirectly confirmed possible reasons why the pandemic affected the dietary consumption among Chinese residents through heterogeneity analysis, we were not able to directly attribute the total effects of the pandemic on people’s dietary consumption due to data limitations, such as the income effect, health awareness effect, food supply and prices, and other behavioral changes (e.g., physical activity and cooking). Third, given that the newest data used in this study were recorded in 2020, the main findings of the current research can only reflect the short-term effects of the pandemic on people’s dietary consumption. The long-term impacts of the pandemic remain to be explored. Therefore, our findings should be interpreted and generalized with caution. More relevant studies regarding the behavioral changes induced by the COVID-19 pandemic are encouraged.

## 5. Conclusions

This study empirically examined the impacts of the COVID-19 pandemic on dietary consumption among Chinese residents. The results suggest that the pandemic increased the number of categories of food consumption as a behavioral change to cope with risk and uncertainty during the pandemic. Meanwhile, we also found that residents tended to have a healthier diet, such as increasing consumption of food with high protein (e.g., eggs and dairy) and consuming less food with low-fat content (e.g., red meat) after the outbreak of the pandemic. In addition, the dietary consequences of the pandemic were more pronounced for rural areas, eastern region, regions with a high old-age dependency ratio and a low illiterate rate, and those who had low levels of food consumption. Despite the positive effects on dietary consumption of the pandemic, our study highlighted that the publicity of a healthy diet, the improvement of social security programs, and the promotion of logistic systems should not be neglected, especially for underdeveloped areas and regions with a high proportion of the elderly and low-educated populations.

## Figures and Tables

**Table 1:** Descriptive statistics.

Variable	(1) Overall		(2) 2015~2019		(3) 2020		Diff. (3)-(2)
	Mean	S.D.	Mean	S.D.	Mean	S.D.	*t*-Test
Urban food consumption (log)							
Grain	4.735	0.155	4.725	0.155	4.787	0.146	0.062 **
Red meat	3.362	0.243	3.372	0.246	3.316	0.228	−0.056
Poultry	2.238	0.457	2.205	0.450	2.404	0.463	0.199 **
Aquatic products	2.445	0.655	2.434	0.651	2.500	0.682	0.066
Eggs	2.400	0.320	2.370	0.305	2.550	0.356	0.180 ***
Dairy	2.868	0.354	2.863	0.352	2.893	0.367	0.030
Vegetables	4.645	0.141	4.639	0.141	4.676	0.137	0.037
Fruits	4.081	0.296	4.068	0.296	4.145	0.290	0.077
Rural food consumption (log)							
Grain	5.055	0.179	5.043	0.182	5.115	0.151	0.072 **
Red meat	3.171	0.321	3.186	0.327	3.093	0.281	−0.093
Poultry	2.000	0.672	1.942	0.653	2.288	0.700	0.346 ***
Aquatic products	1.889	0.910	1.855	0.901	2.057	0.952	0.202
Eggs	2.158	0.462	2.116	0.440	2.367	0.520	0.251 ***
Dairy	2.057	0.517	2.051	0.529	2.091	0.463	0.040
Vegetables	4.444	0.326	4.429	0.338	4.521	0.250	0.092
Fruits	3.576	0.539	3.552	0.547	3.699	0.482	0.147
Measures of COVID-19 (log)							
Number of confirmed cases	1.015	2.374	0.000	0.000	6.093	1.674	6.093 ***
Number of suspect cases	0.430	1.321	0.000	0.000	2.578	2.245	2.578 ***
Number of dead cases	0.253	0.835	0.000	0.000	1.521	1.519	1.521 ***
Control variables							
Per capita GDP (log)	11.046	10.268	11.019	10.244	11.167	10.352	0.148 *
Proportion of primary industry (%)	9.834	9.052	9.888	9.627	9.565	5.430	−0.323
Child dependency ratio (%)	23.686	6.565	23.265	6.435	25.790	6.912	2.525 *
Old-age dependency ratio (%)	15.716	3.846	15.093	3.434	18.832	4.315	3.739 ***
Illiteracy rate (%)	5.803	6.048	6.107	6.212	4.283	4.962	−1.824

*Notes:* S.D., standard deviation, * *p* < 0.1, ** *p* < 0.05, *** *p* < 0.01.

**Table 2:** Impacts of COVID-19 on urban and rural dietary consumption.

	(1)	(2)	(3)	(4)	(5)	(6)	(7)	(8)
	Grain	Red Meat	Poultry	Aquatic Products	Eggs	Dairy	Vegetables	Fruits
*Panel A: Urban areas*								
Number of confirmed cases	0.015 ***	−0.011 **	0.016 **	−0.001	0.022 ***	0.010 **	0.006	−0.005
	(0.004)	(0.004)	(0.006)	(0.005)	(0.004)	(0.005)	(0.004)	(0.004)
Per capita GDP	0.026 ***	0.012	−0.010	−0.006	−0.006	−0.002	0.008	0.007
	(0.009)	(0.010)	(0.009)	(0.010)	(0.006)	(0.010)	(0.010)	(0.009)
Proportion of primary industry	0.001 ***	0.001 ***	0.001 ***	−0.000	0.002 ***	−0.001 ***	−0.000	−0.001 **
	(0.000)	(0.000)	(0.000)	(0.000)	(0.000)	(0.000)	(0.000)	(0.000)
Child dependency ratio	0.003	0.002	0.006	−0.001	0.002	0.004	0.008	−0.002
	(0.005)	(0.007)	(0.007)	(0.005)	(0.004)	(0.006)	(0.007)	(0.004)
Old-age dependency ratio	−0.000	−0.005	0.007	0.020 **	0.005	−0.015 *	−0.009	0.009
	(0.006)	(0.010)	(0.010)	(0.010)	(0.008)	(0.008)	(0.008)	(0.007)
Illiteracy rate	0.002	−0.012	0.013 **	0.007	0.021 ***	0.008	−0.011	0.002
	(0.006)	(0.011)	(0.005)	(0.007)	(0.005)	(0.010)	(0.016)	(0.008)
Constant	4.529 ***	3.387 ***	1.871 ***	2.128 ***	2.101 ***	2.949 ***	4.608 ***	3.868 ***
	(0.110)	(0.129)	(0.191)	(0.166)	(0.127)	(0.123)	(0.140)	(0.150)
Provincial fixed effects	Yes	Yes	Yes	Yes	Yes	Yes	Yes	Yes
Year fixed effects	Yes	Yes	Yes	Yes	Yes	Yes	Yes	Yes
*R* ^2^	0.280	0.125	0.602	0.275	0.631	0.076	0.124	0.373
*Panel B: Rural areas*								
Number of confirmed cases	0.022 ***	−0.017 *	0.028 ***	0.013 ***	0.029 ***	0.020 **	0.009	−0.007
	(0.004)	(0.009)	(0.005)	(0.004)	(0.005)	(0.009)	(0.007)	(0.004)
Per capita GDP	−0.002	−0.005	−0.012	−0.001	−0.001	0.012	0.021 *	0.027 **
	(0.013)	(0.010)	(0.008)	(0.013)	(0.011)	(0.013)	(0.012)	(0.011)
Proportion of primary industry	−0.000	0.000	−0.001 **	0.000	0.000	−0.001 **	0.001 **	0.006 ***
	(0.000)	(0.000)	(0.000)	(0.000)	(0.001)	(0.000)	(0.001)	(0.000)
Child dependency ratio	−0.017 **	−0.017	0.004	−0.011 *	−0.003	−0.015	0.013	−0.003
	(0.007)	(0.011)	(0.008)	(0.006)	(0.007)	(0.011)	(0.014)	(0.008)
Old-age dependency ratio	0.018 *	0.006	−0.000	0.009	0.017	0.025	−0.014	0.006
	(0.010)	(0.009)	(0.008)	(0.011)	(0.012)	(0.024)	(0.015)	(0.012)
Illiteracy rate	0.029 **	0.037 **	0.019 **	0.016 **	0.031 ***	0.060 *	−0.017	−0.020
	(0.011)	(0.017)	(0.007)	(0.007)	(0.010)	(0.035)	(0.027)	(0.019)
Constant	5.034 ***	3.211 ***	1.675 ***	1.795 ***	1.706 ***	1.557 ***	4.304 ***	3.340 ***
	(0.177)	(0.173)	(0.173)	(0.172)	(0.198)	(0.352)	(0.224)	(0.176)
Provincial fixed effects	Yes	Yes	Yes	Yes	Yes	Yes	Yes	Yes
Year fixed effects	Yes	Yes	Yes	Yes	Yes	Yes	Yes	Yes
*R* ^2^	0.345	0.295	0.802	0.679	0.636	0.264	0.186	0.668

*Notes:* Standard errors in parentheses are clustered at the provincial level. * *p* < 0.1, ** *p* < 0.05, *** *p* < 0.01

**Table 3:** Impacts of COVID-19 on urban and rural dietary consumption: heterogeneity by region.

	(1)	(2)	(3)	(4)	(5)	(6)	(7)	(8)
	Grain	Red Meat	Poultry	Aquatic Products	Eggs	Dairy	Vegetables	Fruits
*Panel A: Urban areas*								
Number of confirmed cases (Eastern region)	0.015 ***	−0.010	0.014 ***	−0.003	0.017 ***	0.020 ***	0.006	−0.009
	(0.005)	(0.009)	(0.005)	(0.005)	(0.003)	(0.005)	(0.005)	(0.006)
*R* ^2^	0.419	0.241	0.792	0.359	0.822	0.329	0.324	0.590
Number of confirmed cases (Central region)	0.015 *	−0.005	0.006	−0.015	0.013	−0.003	0.008	−0.005
	(0.007)	(0.007)	(0.006)	(0.008)	(0.008)	(0.006)	(0.008)	(0.008)
*R* ^2^	0.734	0.393	0.784	0.519	0.806	0.198	0.264	0.833
Number of confirmed cases (Western region)	0.010	−0.010	0.001	−0.012	0.016	0.003	0.007	0.003
	(0.013)	(0.006)	(0.018)	(0.014)	(0.014)	(0.012)	(0.007)	(0.009)
*R* ^2^	0.127	0.376	0.332	0.329	0.369	0.164	0.300	0.170
*Panel B: Rural areas*								
Number of confirmed cases (Eastern region)	0.023 ***	−0.026 ***	0.025 ***	0.007	0.021 ***	0.020 **	0.012 ***	−0.001
	(0.005)	(0.007)	(0.008)	(0.005)	(0.004)	(0.009)	(0.004)	(0.005)
*R* ^2^	0.391	0.374	0.843	0.747	0.838	0.520	0.344	0.726
Number of confirmed cases (Central region)	0.016	−0.011	0.023 *	−0.003	0.020 *	−0.009	0.019	−0.019 *
	(0.011)	(0.010)	(0.011)	(0.009)	(0.011)	(0.008)	(0.012)	(0.009)
*R* ^2^	0.661	0.467	0.872	0.887	0.835	0.733	0.448	0.874
Number of confirmed cases (Western region)	0.010	−0.003	0.022 **	0.005	0.019	0.018	0.015	−0.004
	(0.010)	(0.023)	(0.008)	(0.005)	(0.017)	(0.044)	(0.011)	(0.009)
*R* ^2^	0.519	0.305	0.730	0.679	0.346	0.448	0.426	0.689

*Notes:* Standard errors in parentheses are clustered at the provincial level. All regressions included control variables, including per capita GDP, proportion of output value of the primary industry, child support ratio, old-age dependency ratio, and illiteracy rate. Provincial fixed effects and year fixed effects are also controlled in the regressions. * *p* < 0.1, ** *p* < 0.05, *** *p* < 0.01.

**Table 4:** Impacts of COVID-19 on urban and rural dietary consumption: heterogeneity by old-age dependency ratio.

	(1)	(2)	(3)	(4)	(5)	(6)	(7)	(8)
	Grain	Red Meat	Poultry	Aquatic Products	Eggs	Dairy	Vegetables	Fruits
*Panel A: Urban areas*								
Number of confirmed cases (high old-age dependency ratio)	0.022 ***	−0.023 ***	0.019 ***	−0.003	0.024 ***	0.016 ***	0.014 ***	0.007 *
	(0.005)	(0.008)	(0.004)	(0.002)	(0.003)	(0.005)	(0.005)	(0.004)
*R* ^2^	0.479	0.308	0.630	0.492	0.810	0.218	0.255	0.643
Number of confirmed cases (low old-age dependency ratio)	0.008	−0.001	0.004	−0.007	0.010	0.001	0.007	−0.006
	(0.013)	(0.004)	(0.019)	(0.017)	(0.011)	(0.008)	(0.007)	(0.012)
*R* ^2^	0.158	0.254	0.380	0.095	0.264	0.175	0.326	0.166
*Panel B: Rural areas*								
Number of confirmed cases (high old-age dependency ratio)	0.023 ***	−0.021 ***	0.032 ***	0.007	0.029 ***	0.007	0.013 **	0.007
	(0.006)	(0.004)	(0.011)	(0.004)	(0.006)	(0.005)	(0.005)	(0.004)
*R* ^2^	0.405	0.517	0.653	0.758	0.762	0.367	0.314	0.781
Number of confirmed cases (low old-age dependency ratio)	0.014 ***	−0.009	0.022 ***	0.015	0.009	−0.011	0.015	−0.007
	(0.005)	(0.024)	(0.006)	(0.011)	(0.013)	(0.026)	(0.017)	(0.011)
*R* ^2^	0.380	0.308	0.832	0.499	0.464	0.433	0.291	0.566

*Notes:* Standard errors in parentheses are clustered at the provincial level. All regressions included control variables, including per capita GDP, proportion of output value of the primary industry, child support ratio, old-age dependency ratio, and illiteracy rate. Provincial fixed effects and year fixed effects are also controlled in the regressions. * *p* < 0.1, ** *p* < 0.05, *** *p* < 0.01.

**Table 5:** Impacts of COVID-19 on urban and rural dietary consumption: heterogeneity by illiteracy rate.

	(1)	(2)	(3)	(4)	(5)	(6)	(7)	(8)
	Grain	Red Meat	Poultry	Aquatic Products	Eggs	Dairy	Vegetables	Fruits
*Panel A: Urban areas*								
Number of confirmed cases (high illiteracy rate)	0.003	−0.005	0.006	−0.003	0.014	−0.003	−0.002	0.002
	(0.008)	(0.006)	(0.014)	(0.011)	(0.009)	(0.008)	(0.005)	(0.008)
*R* ^2^	0.111	0.124	0.382	0.252	0.291	0.151	0.234	0.286
Number of confirmed cases (low illiteracy rate)	0.020 ***	−0.020 ***	0.019 ***	−0.005	0.020 ***	0.017 ***	0.008	−0.010
	(0.006)	(0.005)	(0.005)	(0.007)	(0.004)	(0.005)	(0.005)	(0.006)
*R* ^2^	0.497	0.242	0.710	0.323	0.804	0.206	0.238	0.631
*Panel B: Rural areas*								
Number of confirmed cases (high illiteracy rate)	0.013 *	−0.008	0.021 ***	0.004	0.018	−0.013	0.001	−0.011
	(0.007)	(0.022)	(0.007)	(0.006)	(0.011)	(0.015)	(0.010)	(0.009)
*R* ^2^	0.390	0.336	0.676	0.572	0.297	0.353	0.362	0.696
Number of confirmed cases (low illiteracy rate)	0.019 ***	−0.022 ***	0.031 ***	0.010 **	0.021 ***	0.006	0.010	−0.010
	(0.005)	(0.005)	(0.006)	(0.004)	(0.004)	(0.008)	(0.006)	(0.006)
*R* ^2^	0.516	0.344	0.836	0.795	0.827	0.296	0.348	0.691

*Notes:* Standard errors in parentheses are clustered at the provincial level. All regressions included control variables, including per capita GDP, proportion of output value of the primary industry, child support ratio, old-age dependency ratio, and illiteracy rate. Provincial fixed effects and year fixed effects are also controlled in the regressions. * *p* < 0.1, ** *p* < 0.05, *** *p* < 0.01.

**Table 6:** Results of quantile regressions for panel data.

	(1)		(2)		(3)		(4)		(5)	
	Q (10)		Q (25)		Q (50)		Q (75)		Q (90)	
*Panel A: Urban areas*										
Grain	0.017 ***	(0.006)	0.017 ***	(0.004)	0.015 ***	(0.003)	0.014 ***	(0.005)	0.013 **	(0.006)
Red meat	−0.015	(0.015)	−0.013	(0.012)	−0.011	(0.007)	−0.009	(0.006)	−0.008	(0.008)
Poultry	0.020 **	(0.008)	0.018 ***	(0.006)	0.016 ***	(0.004)	0.014 **	(0.006)	0.012	(0.008)
Aquatic products	0.004	(0.006)	0.002	(0.004)	−0.001	(0.004)	−0.005	(0.005)	−0.008	(0.007)
Eggs	0.023 ***	(0.007)	0.022 ***	(0.005)	0.022 ***	(0.003)	0.021 ***	(0.004)	0.021 ***	(0.006)
Dairy	0.012	(0.008)	0.011 *	(0.006)	0.010 **	(0.004)	0.010	(0.006)	0.009	(0.008)
Vegetables	0.007	(0.009)	0.007	(0.005)	0.006	(0.013)	0.006	(0.023)	0.006	(0.028)
Fruits	−0.004	(0.012)	−0.005	(0.008)	−0.005	(0.008)	−0.005	(0.015)	−0.005	(0.022)
*Panel B: Rural areas*										
Grain	0.025 ***	(0.008)	0.024 ***	(0.006)	0.022 ***	(0.004)	0.020 ***	(0.005)	0.019 ***	(0.007)
Red meat	−0.020	(0.015)	−0.019	(0.012)	−0.017 **	(0.008)	−0.015 *	(0.009)	−0.013	(0.014)
Poultry	0.030 ***	(0.008)	0.030 ***	(0.006)	0.028 ***	(0.005)	0.027 ***	(0.006)	0.026 ***	(0.009)
Aquatic products	0.020 ***	(0.006)	0.018 ***	(0.004)	0.014 ***	(0.004)	0.009	(0.008)	0.006	(0.011)
Eggs	0.030 ***	(0.009)	0.030 ***	(0.007)	0.029 ***	(0.005)	0.029 ***	(0.007)	0.028 ***	(0.009)
Dairy	0.022 *	(0.013)	0.021 **	(0.009)	0.020 **	(0.008)	0.019 *	(0.011)	0.018	(0.017)
Vegetables	0.009	(0.013)	0.009	(0.009)	0.009	(0.007)	0.009	(0.009)	0.008	(0.013)
Fruits	−0.002	(0.008)	−0.004	(0.006)	−0.007	(0.004)	−0.009	(0.006)	−0.011	(0.008)

*Notes:* Standard errors in parentheses are clustered at the provincial level. All regressions included control variables, including per capita GDP, proportion of output value of the primary industry, child support ratio, old-age dependency ratio, and illiteracy rate. Provincial fixed effects and year fixed effects are also controlled in the regressions. * *p* < 0.1, ** *p* < 0.05, *** *p* < 0.01.

**Table 7:** Robustness checks: different measures of COVID-19.

	(1)	(2)	(3)	(4)	(5)	(6)	(7)	(8)
	Grain	Red Meat	Poultry	Aquatic Products	Eggs	Dairy	Vegetables	Fruits
*Panel A: Urban areas*								
Number of suspect cases	0.016 ***	−0.010	0.019 **	−0.004	0.024 ***	0.014 **	0.006	−0.007
	(0.005)	(0.008)	(0.007)	(0.004)	(0.006)	(0.006)	(0.006)	(0.005)
*R* ^2^	0.221	0.098	0.585	0.276	0.582	0.070	0.115	0.373
Number of dead cases	0.035 ***	−0.011	0.021 *	−0.000	0.028 **	0.013	0.016 **	−0.009
	(0.008)	(0.013)	(0.012)	(0.007)	(0.013)	(0.010)	(0.008)	(0.006)
*R* ^2^	0.282	0.087	0.569	0.274	0.548	0.040	0.130	0.371
*Panel B: Rural areas*								
Number of suspect cases	0.026 ***	−0.019 **	0.027 ***	0.015 ***	0.035 ***	0.022 **	0.014	−0.002
	(0.009)	(0.007)	(0.009)	(0.005)	(0.011)	(0.010)	(0.009)	(0.006)
*R* ^2^	0.295	0.277	0.768	0.670	0.602	0.277	0.190	0.663
Number of dead cases	0.031 *	−0.031 **	0.041 *	0.030 **	0.034 *	0.024 **	0.018	−0.004
	(0.018)	(0.013)	(0.022)	(0.014)	(0.020)	(0.011)	(0.012)	(0.008)
*R* ^2^	0.246	0.278	0.766	0.678	0.555	0.267	0.182	0.663

*Notes:* Standard errors in parentheses are clustered at the provincial level. All regressions included control variables, including per capita GDP, proportion of output value of the primary industry, child support ratio, old-age dependency ratio, and illiteracy rate. Provincial fixed effects and year fixed effects are also controlled in the regressions. * *p* < 0.1, ** *p* < 0.05, *** *p* < 0.01.

## Data Availability

The data from *China Statistical Yearbook* can be accessed through the official website (http://www.stats.gov.cn/tjsj/ndsj/ (accessed on 21 March 2022)).

## Appendix A

**Table A1:** Robustness checks: food price as an additional control variable.

	(1)	(2)	(3)	(4)	(5)	(6)	(7)	(8)
	Grain	Red Meat	Poultry	Aquatic Products	Eggs	Dairy	Vegetables	Fruits
*Panel A: Urban areas*								
Number of confirmed cases	0.015 ***	−0.013 **	0.018 **	0.001	0.013 **	0.010 **	0.007	−0.005
	(0.004)	(0.005)	(0.007)	(0.005)	(0.005)	(0.005)	(0.004)	(0.004)
Food price	−0.001	−0.002 *	0.001	−0.006 ***	−0.004 ***	−0.006 ***	−0.001	−0.000
	(0.005)	(0.001)	(0.001)	(0.002)	(0.001)	(0.002)	(0.001)	(0.001)
*R* ^2^	0.280	0.130	0.604	0.295	0.669	0.107	0.127	0.373
*Panel B: Rural areas*								
Number of confirmed cases	0.022 ***	−0.016 *	0.029 ***	0.015 ***	0.030 ***	0.021 ***	0.008	−0.007
	(0.004)	(0.009)	(0.005)	(0.004)	(0.005)	(0.007)	(0.007)	(0.005)
Food price	−0.003	−0.003	−0.001	−0.007 **	−0.006 ***	−0.007 **	−0.001	0.000
	(0.006)	(0.002)	(0.001)	(0.003)	(0.001)	(0.003)	(0.001)	(0.001)
*R* ^2^	0.346	0.297	0.802	0.691	0.684	0.274	0.188	0.668

*Notes:* Standard errors in parentheses are clustered at the provincial level. All regressions included control variables, including per capita GDP, proportion of output value of the primary industry, child support ratio, old-age dependency ratio, and illiteracy rate. Provincial fixed effects and year fixed effects are also controlled in the regressions. Food price was measured using the food price index (i.e., current food price is divided by that of last year, then multiplied by 100). The food price variable for each column was different depending on the category of food examined. * *p* < 0.1, ** *p* < 0.05, *** *p* < 0.01.
